# Synthesis of Sulfur-Selenium Doped Carbon Quantum Dots for Biological Imaging and Scavenging Reactive Oxygen Species

**DOI:** 10.1038/s41598-019-55996-w

**Published:** 2019-12-23

**Authors:** Guojie Huang, Yaqi Lin, Linxiu Zhang, Zhihong Yan, Yudong Wang, Yi Liu

**Affiliations:** 1 The First Affiliated Hospital of Guangdong Pharmaceutical University, Guangdong, China; 2 College of Pharmacy, Guangdong Pharmaceutical University, Guangdong, China; 3 School of Chemistry and Chemical Engineering,Guangdong Pharmaceutical University, Guangdong, China

**Keywords:** Biochemistry, Chemical biology, Chemistry, Materials science, Nanoscience and technology

## Abstract

The sulfur-selenium doped carbon quantum dots (S,Se-CQDs) were synthesized by one-step through hydrothermal method in this study, which have high fluorescence quantum yield (43%) and advanced ability to scavenge reactive oxygen species (ROS). They were characterized by transmission electron microscope (TEM), nuclear magnetic resonance (NMR), X-ray photoelectron spectroscopy (XPS), fourier transform infrared spectroscopy (FTIR). The results showed that the clearance rate of free radical reached to 40% with 200 μg/mL of S,Se-CQDs. The antioxidant activity of S,Se-CQDs is related to -SH and Se-SH on carbon quantum dots. S,Se-CQDs were able to access to cells which is beneficial to enhance the removal efficiency to ROS. In the biocompatibility experiment, the cell survival rate exceeded 95%, there was little effect on hatching rate, survival rate and heart rate of zebrafish which demonstrated that S,Se-CQDs have an excellent biocompatibility. It prompts that S,Se-CQDs will has proud application prospects in the field of biomedicine.

## Introduction

ROS are the by-product of aerobic respiration^1^. ROS in organism include hydrogen peroxide (H_2_O_2_), hydroxyl radicals (·OH) and superoxide anions (O_2_·^−^)^2^. Excessive ROS can damage cells through hurt DNA, proteins and lipids directly^3^, then induces a series of physiological and pathological effects. The guanine nucleotides were oxidated by ROS and then doped into DNA or RNA can induce cell death^4^.

CQDs, as a new member of the carbon material family, has been concerned widely due to their excellent optical and chemical properties^5,6^. CQDs have been applied in many application fields: photoconductor^7^, bioimaging^8,9^, biosensing^10,11^, drug carriers^12–14^ and information confidentiality^15,16^. Carbon quantum dots with antioxidant activity have attracted wide attention of researchers. Avinash^17^ adopted a coconut shell to synthesize water-dispersible CQDs by hydrothermal method, the DPPH test proved that the CQDs had excellent scavenging effect on free radicals. Li^18^ adopted citric acid and selenocysteine to synthesize Se-CQDs by hydrothermal and certificated that the Se-CQDs have outstanding scavenging ability to H_2_O_2_ and hydroxyl radicals *in vitro*. There are many substances to scavenge ROS in our body, like reduced glutathione, plays an important role in anti-oxidation because of sulfhydry^19^. Considering to synthesize a S,Se-CQDs with outstanding biocompatibility and predominant scavenging ability to ROS may be regarded as a good idea to regulate the ROS level in organisms.

In this work, we prepared S,Se-CQDs through hydrothermal method. Experimental results showed that the S,Se-CQDs possessed excellent clearing ability to ROS. S,Se-CQDs had an outstanding biocompatibility, the cell survival rate exceeded 95% in cell toxicity experiments and had little effect to zebrafish. S,Se-CQDs can pass through the cell membrane and distributed in cytoplasm which is the key to improve the efficiency of clearing ROS.

## Results and Discussion

### Synthesis and characterization of S,Se-CQDs

**Figure 1:**
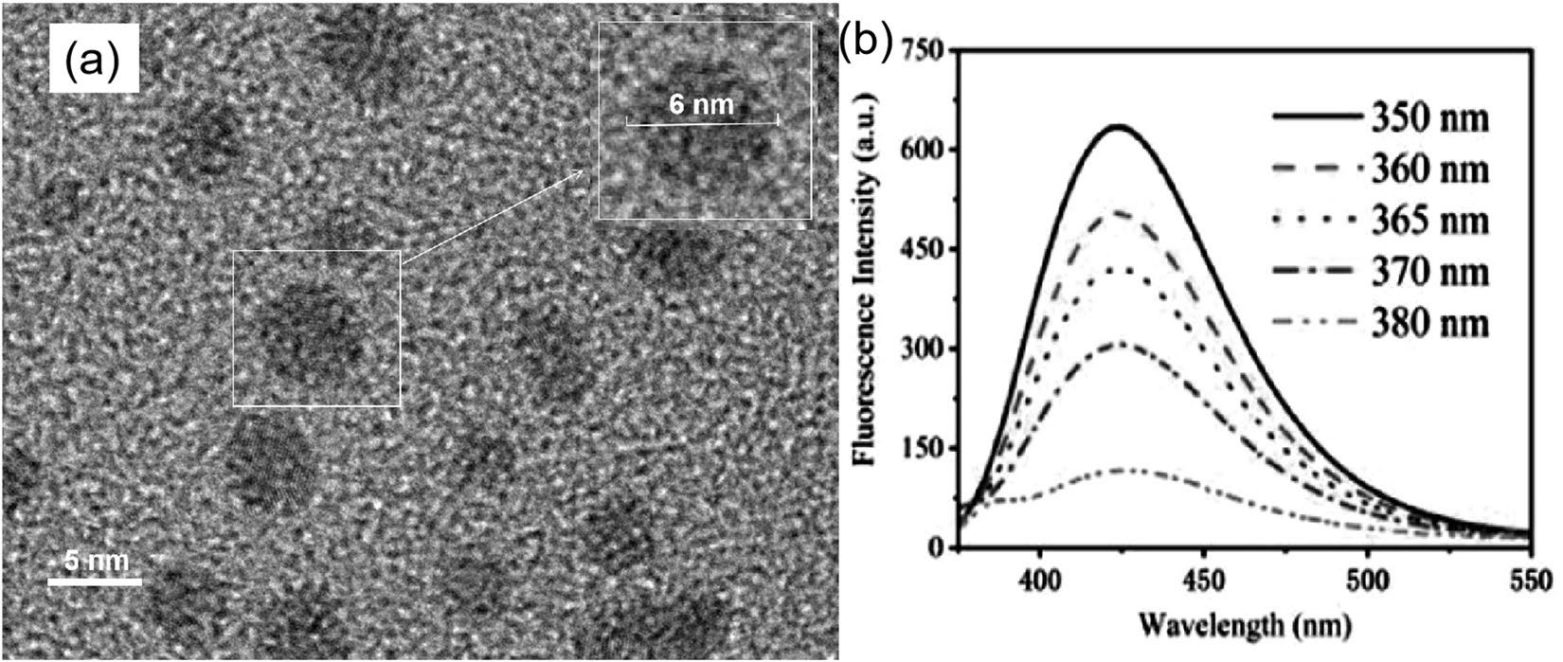
TEM image of S,Se-CQDs (**a**) and PL spectrum of S,Se-CQDs with excitation of different wavelengths (**b**).

The TEM image (Fig. 1a) show that the morphology of S,Se-CQDs are approximately spherical, the average diameter is about 6 nm. The fluorescence quantum yield of S,Se-CQDs reach to 43%, measuring fluorescence intensity at different excitation wavelengths. The photoluminescence of S,Se-CQDs is not related to the excitation wavelength (Fig. 1b).

**Figure 2:**
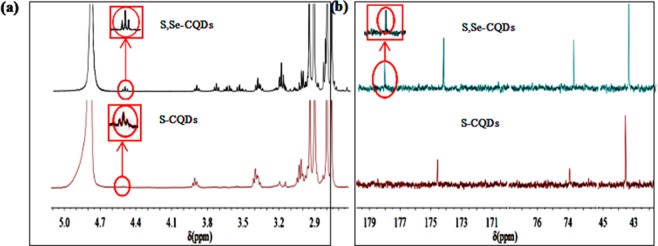
(**a**) ^1^H NMR spectrum of CQDs (**a**), and ^13^C NMR spectrum of CQDs (**b**).

S,Se-CQDs were characterized by NMR (Fig. 2). The ^1^H NMR spectrum of S-CQDs and S,Se-CQDs showed the signals at about 4.5 ppm belonging to sulfhydryl. The ^13^C NMR spectrum of S,Se-CQDs has one more signal than that of S-CQDs at about 180 ppm should correspond to C-Se^15^.

**Figure 3:**
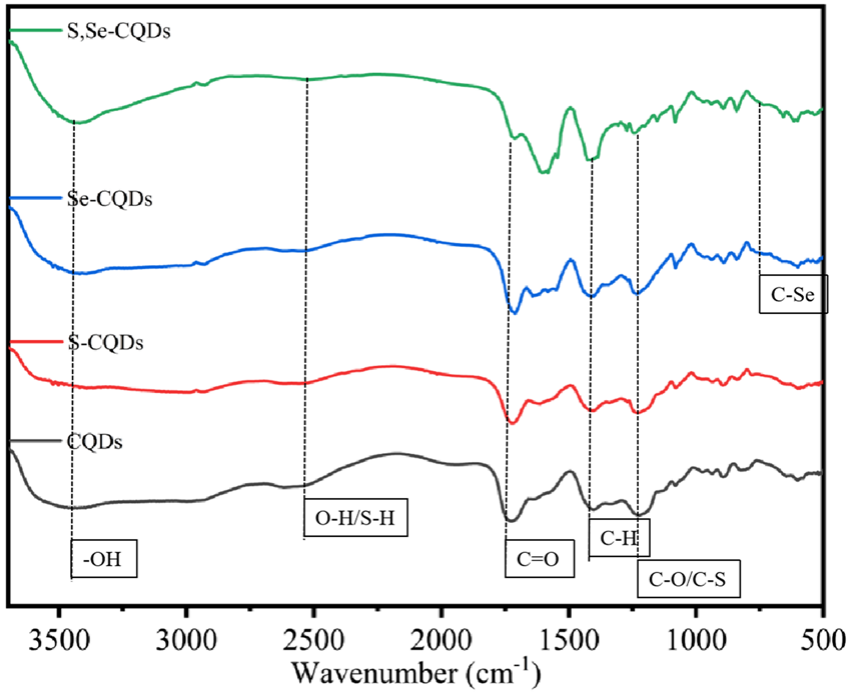
FTIR spectrum of different CQDs.

The structure of S,Se-CQDs was further studied by FTIR (Fig. 3). In the spectrum of Se-CQDs and S,Se-CQDs, the absorption bands of C-Se at about 750 cm^−1^ confirms the doping of Se in the S,Se-CQDs. The peak at 1200–1300 cm^−1^ is the absorption of C-O/C-S^20^, the weak absorption peak at 2500–2600 cm^−1^ indicated there is -OH/-SH on the surface of S,Se-CQDs.

**Figure 4:**
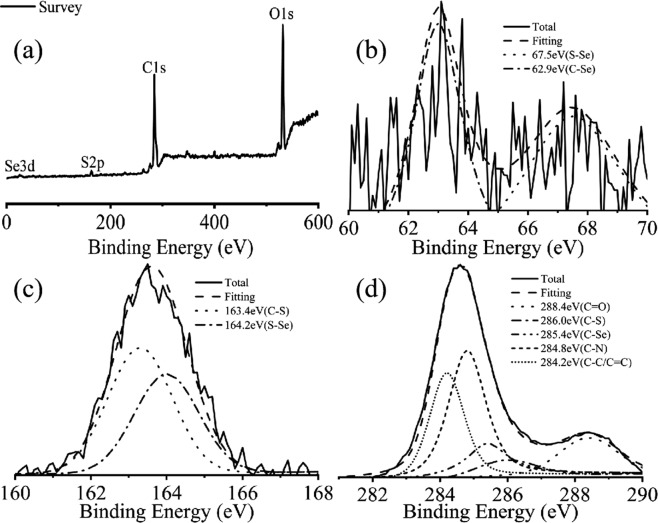
XPS spectrum of S,Se-CQDs (**a**), High resolution map of Se3d (**b**), High resolution map of S2p (**c**) and High resolution map of C1s (**d**).

The elemental composition of S,Se-CQDs was identified by XPS. Figure 4a show that S,Se-CQDs are mainly composed of carbon, oxygen, selenium and sulfur. The C1s spectrum (Fig. 4d) show five peaks after fitting, C=O (288.4 eV), C-S (286.0 eV), C-Se (285.4 eV), C-N (284.8 eV) and C-C (284.2 eV). Binding to the peaks of S-Se (67.1 eV) and C-Se (63.1 eV) in the Se3d spectrum (Fig. 4b) and the peaks of C-S (163.1 eV) and S-Se (164.1 eV) in the S2p spectrum (Fig. 4c), proven sulfur and selenium have introduced on carbon quantum dots successfully^18,21^, they are presumed to exist in the form of -SH and Se-SH.

### Antioxidant of S,Se-CQDs

**Figure 5:**
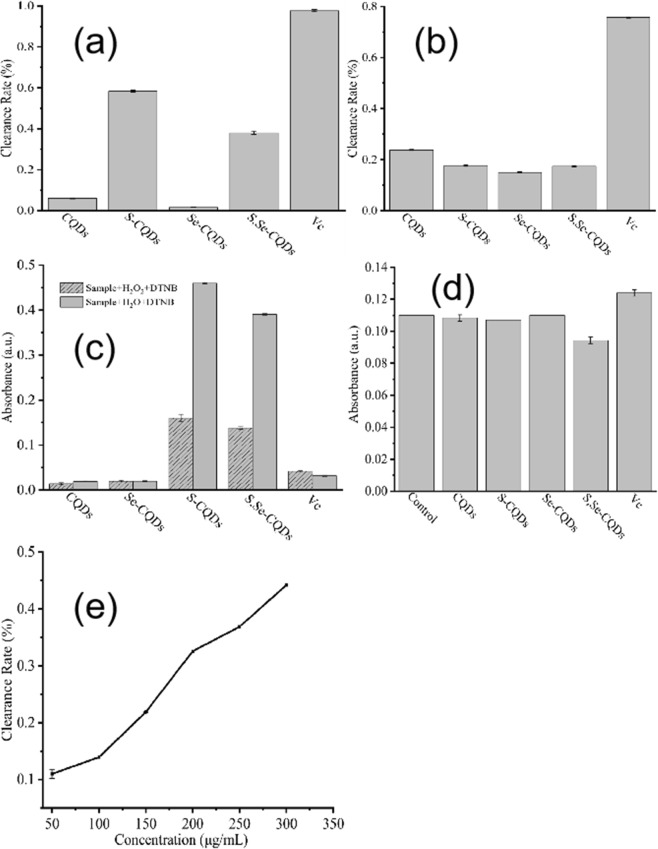
Study on the antioxidant capacity of S,Se-CQDs: DPPH test (**a**),·OH removal test (**b**), H_2_O_2_ removal test (**c**), O_2_·^−^ removal test (**d**) and Relationship between DPPH free radical scavenging rate and concentration of S,Se-CQDs (**e**).

DPPH is a very stable nitrogen-centered free radical that can captures (“clears”) other free radicals^22^. DPPH radicals have strong absorption peaks at ultraviolet 517 nm UV. The results(Fig. 5a) showed the scavenging ability to free radical of S-CQDs and S,Se-CQDs are better than CQDs and Se-CQDs which may be related to the sulfhydryl on CQDs. S,Se-CQDs have a positive correlation between free radical scavenging ability and concentration (Fig. 5e).

The hydroxyl radical (·OH) was produced by the Fenton reaction system^23^. Hydroxyl radical reacts with salicylic acid to form 2,3-dihydroxybenzoic acid with special absorption at 510 nm UV^24^.The results (Fig. 5b) showed the ability of S,Se-CQDs to clear ·OH reached to 15%, because the sulfhydryl on the surface of S,Se-CQDs can provide H to react with ·OH which endue S,Se-CQDs a certain ability to scavenge ·OH^25^.

DTNB react with thiol-containing compounds to break disulfide bonds to form 2-nitro-5-thiobenzoic acid (NBT^−^), than the NBT^2−^ is formed after ionization at neutral or weak alkaline conditions which has the maximum absorption at 412 nm UV^26^. According to the changes of the absorbance value added H_2_O_2_ or not in the reaction system, the clearing ability of S,Se-CQDs can be reflected. The results showed (Fig. 5c) S,Se-CQDs has higher absorbance value when no H_2_O_2_ was added. After adding H_2_O_2_, the absorbance values decreased significantly. Because sulfhydryl can react with H_2_O_2_^27^, the amount of sulfhydryl on S,Se-CQDs determines its ability to scavenge H_2_O_2_.

The O_2_·^−^ was produced by the AP-TEMED system. XTT can be oxidized by O_2_·^-^ to form water-soluble XTT-formamidine with maximum absorption at 470 nm^28,29^. Comparing the change in absorbance can reflect the ability of the sample to remove O_2_·^−^. The results (Fig. 5d) showed that the absorbance values of S-CQDs and S,Se-CQDs had decreased compared with the control group, explained both of them had reacted with O_2_·^−^, S,Se-CQDs is lower than S-CQDs, but Se-CQDs almost no change at all, speculated that the scavenging effect on O_2_·^−^ may be the result of the interaction of -SH and Se on the S,Se-CQDs.

The above results proved that S,Se-CQDs has a predominant scavenging effect on ROS. The ability of S,Se-CQDs to scavenge free radicals reached to 40%. S,Se-CQDs had a certain effect on clearing H_2_O_2_, ·OH and O_2_·^−^. Removal of H_2_O_2_ and ·OH was mainly the role of sulfhydryl on S,Se-CQDs, the interaction of -SH and Se might affect the level of O_2_·^−^. The possible reaction modes of S-CQDs or S,Se-CQDs and reactive oxygen species may be as follows:

1 $$2-{\rm{SH}}+{{{\rm{O}}}_{2}}^{.-}+{{\rm{H}}}^{+}\to -{\rm{S}}-{\rm{S}}-+{{\rm{H}}}_{2}{{\rm{O}}}_{2}$$

2 $$-{\rm{S}}-{\rm{S}}-+-{\rm{Se}}\to -{\rm{SH}}+\,-{\rm{S}}-{\rm{Se}}-$$

3 $$2-{\rm{SH}}+{{\rm{H}}}_{2}{{\rm{O}}}_{2}\to -{\rm{S}}-{\rm{S}}-+2\,{{\rm{H}}}_{2}{\rm{O}}$$

4 $$2-{\rm{SH}}+\,2\,\cdot {\rm{OH}}\to -{\rm{S}}-{\rm{S}}-+\,2\,{{\rm{H}}}_{2}{\rm{O}}$$

### Biocompatibility of S,Se-CQDs

**Figure 6:**
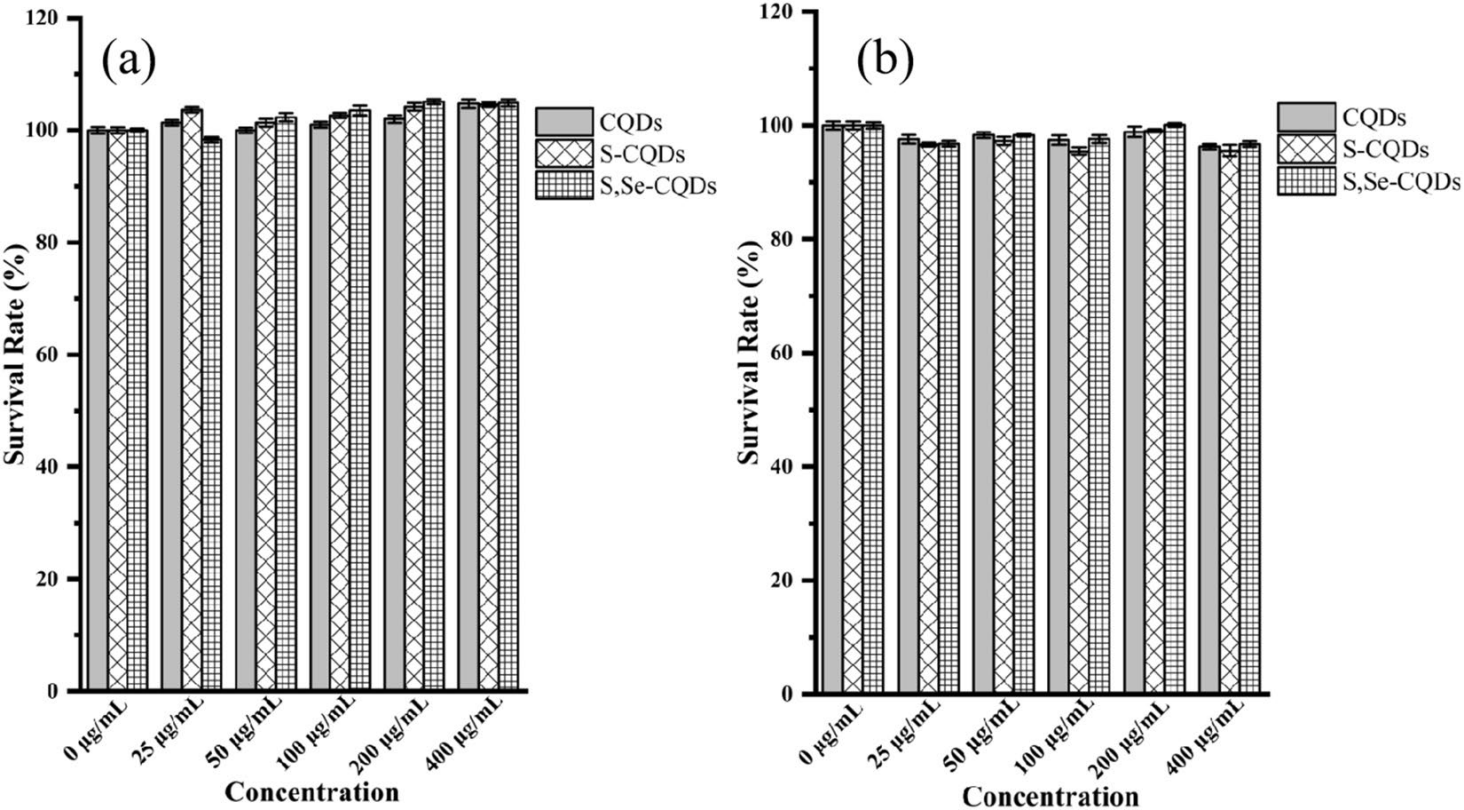
Exploration of Cytotoxicity: Hela cell (**a**) and MDA-MD-231cell (**b**).

The toxicity of S,Se-CQDs was determined by MTT assay^30^. The cell viability of Hela cells and MDA-MB-231 cells after incubated for 24 h with S,Se-CQDs solution(100 μL) maintaining above 95% at a concentration of 400 μg/mL, demonstrated that S,Se-CQDs have excellent biocompatibility (Fig. 6).

**Figure 7:**
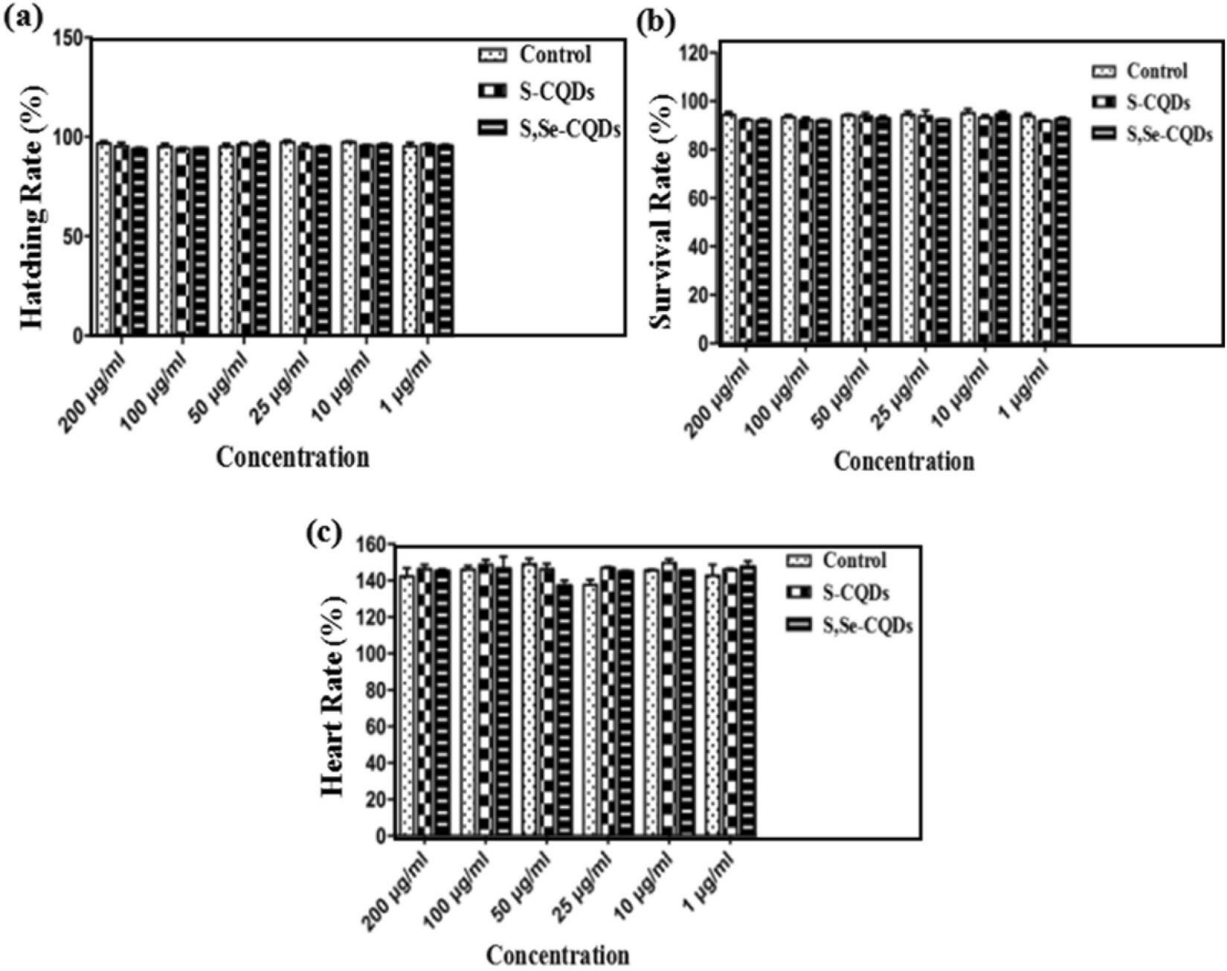
The effect of S,Se-CQDs on zebrafish: Hatching rate of Zebrafish fertilized egg after 72 hours (**a**), Heart rate of zebrafish embryos after 96 h (**b**) and Survival rate of zebrafish embryos after 96 hours (**c**).

The results (Fig. 7a) of zebrafish experiment^31^ showed the hatching rate of Zebrafish fertilized egg has no significant difference after incubating with S,Se-CQDs for 72 h compared with the control group. After a total of 96 h of incubation, S,Se-CQDs did not show apparent difference in survival rate(Fig. 7b) and heart rate (Fig. 7c) of zebrafish embryos compared with the control, further indicated that S,Se-CQDs have outstanding biocompatibility, doping Se has little effect on the biocompatibility of S,Se-CQDs.

### Biological imaging of S,Se-CQDs

**Figure 8:**
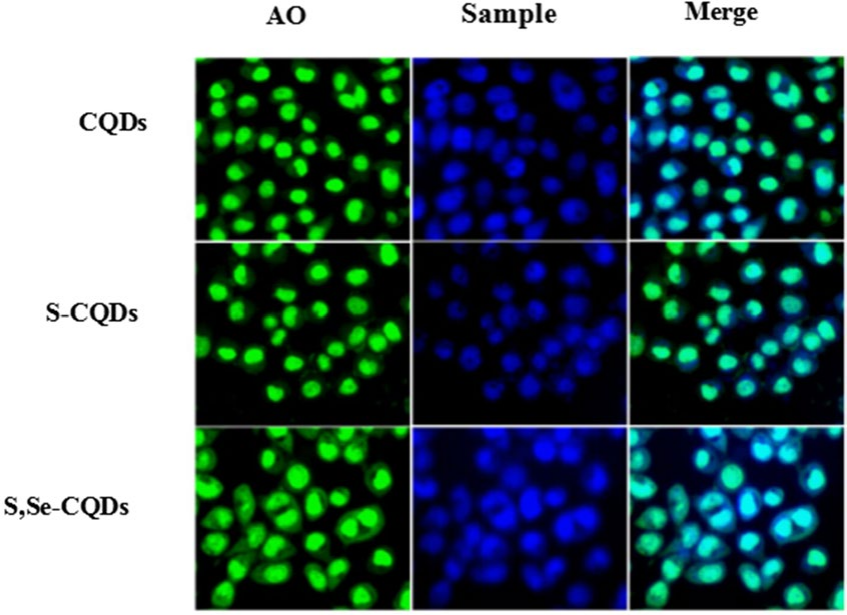
Confocal fluorescence microphotographs of CDs labeled Hela cells at excitation wavelengths of 488 nm (green) and 361 nm (blue).

Acridine orange (AO) has membrane permeability, can penetrate the cell membrane, stains the nuclear DNA or RNA, green fluorescence was emitted after being excited at 488 nm excitation. The blue fluorescence was emitted after being excited at 361 nm excitation of S,Se-CQDs. Fluorescence was observed after incubation with Hela cells for 12 h with AO and S,Se-CQDs respectively, explored the ability of S,Se-CQDs to enter cells and the distribution site. The results (Fig. 8) showed that the green fluorescence could be seen after stained by AO and the blue fluorescence in the cytoplasm of S,Se-CQDs group was stronger than S-CQDs group which indicated that S,Se-CQDs had stronger ability to enter cell than S-CQDs. ROS produced in the cells are mainly distributed in the cytoplasm. Predominant capability to enter cells and enriched in cytoplasm are more beneficial for S,Se-CQDs to play an important role to regulate the levels of ROS in cell.

## Discussion (Conclusions)

In summary, S,Se-CQDs were synthesized by hydrothermal successfully. High fluorescence quantum yield (43%) make S,Se-CQDs possible for biological imaging. The results of antioxidant experiment certificated S,Se-CQDs have advanced clearing ability of ROS. Excellent biocompatibility and more outstanding ability to enter cells than S-CQDs suggested that S,Se-CQDs are more suitable for clearing the ROS from the cytoplasm and as a drug carrier in the biomedical field.

## Methods

### Reagents and apparatus

Citric acid was purchased from Sinopharm Chemical Reagent Co., Ltd. Sodium selenite was purchased from Nanjing Chemical Co., Ltd., hydrogen peroxide was purchased from Jiangsu Qiangsheng Chemical Co., Ltd. Absolute ethanol, salicylic acid was purchased from Tianjin Damao Chemical Reagent Factory. Mercaptoethylamine, 5,5′-dithiobis (2-nitrobenzoic acid) (DTNB) and Sodium 2,3-bis(2-methoxy-4-nitro-5-sulfophenyl)−2H-tetrazolium-5-carboxanilide inner salt (XTT sodium salt) was purchased from Macleans Chemical Reagent Co., Ltd. 1,1-Diphenyl-2-trinitrophenylhydrazine (DPPH), Tris, ammonium persulfate was purchased from Aladdin Reagent Co., Ltd.

TEM was taken with a field-emission transmission electron microscope. Determination of NMR using deuterated water. The PL spectra of S,Se-CQDs were detected using a Fluorescence spectrophotometer. XPS were recorded on an ECSALAB 250 spectrometer. FTIR were detected using a Fourier Transform Infrared Spectrometer.

### Preparation of S,Se-CQDs

Citric acid (5 g), mercaptoethylamine (0.8 g) and sodium selenite (0.06 g) are dissolved in 40 mL of pure water, transfer the solution to 50 mL of PTFE lining, and insert the lining into stainless steel, put the stainless steel reaction kettle in the oven, react at 150 °C for 150 min. Than the reaction solution centrifuges at 8000 r/min for 20 min, separation supernatant and dialysis for 2 days with 200 Da dialysis bag, change water every 5 h, finally freeze-dry. S,Se-CQDs was obtained. The synthesis of CQDs, S-CQDs and Se-CQDs was used citric acid (5 g) or citric acid (5 g) and mercaptoethylamine (0.8 g) or citric acid (5 g) and sodium selenite (0.06 g) as raw materials respectively according to the above method.

### Fluorescence quantum yield

A small amount of standard quinine sulfate (QY = 0.577) was dissolved in a 0.05 N sulfuric acid solution as a reference. Measure the emission peak integral area and UV absorbance (absorbance values need to be kept at 0.05) of the analyte and quinine sulfate at 350 nm (the maximum excitation wavelength of the standard), the quantum yield is calculated as follows:

$${{\rm{Q}}}_{{\rm{s}}}{\rm{Y}}={{\rm{Q}}}_{{\rm{r}}}{\rm{Y}}({{\rm{F}}}_{{\rm{s}}}/{{\rm{F}}}_{{\rm{r}}})({{\rm{A}}}_{{\rm{r}}}/{{\rm{A}}}_{{\rm{s}}}){({{\rm{\eta }}}_{{\rm{s}}}/{{\rm{\eta }}}_{{\rm{r}}})}^{2}$$

QY is the quantum yield, F is the fluorescence emission peak area, A is the absorbance at the excitation wavelength, and η is the refractive index of the solvent. Where s represents the analyte and r represents the reference.

## Antioxidant Experiment

### DPPH test

The experimental design is as follows: control: water (2 mL), DPPH anhydrous ethanol solution (0.4 mM, 2 mL), react in the dark for 30 min; experimental group: sample (200 μg/mL, 2 mL), DPPH anhydrous ethanol solution (0.4 mM, 2 mL), react in the dark for 30 min; blank group: sample (200 μg/mL, 2 mL), absolute ethanol (2 mL), react in the dark for 30 min. The background was calibrated with a mixture of water (2 mL) and absolute ethanol (2 mL), and the absorbance at 517 nm was measured. The free radical scavenging rate is calculated as follows:

$${\rm{Clearance}}\,{\rm{Rate}}( \% )=(1-\frac{A1-A2}{A3})\times 100$$

A1: the absorbance value of the experimental group, A2: the absorbance value of the blank group, A3: the absorbance value of the control.

### ·OH removal test

Control: water (1 mL), FeSO_4_ (1.8 mM, 2 mL), salicylic acid anhydrous ethanol solution (1.8 mM, 1.5 mL), H_2_O_2_ (0.03%, 100 μL) were mixed in a test tube at 37 °C water bath for 30 min; experimental group: sample (200 μg/mL, 1 mL), FeSO_4_ (1.8 mM, 2 mL), salicylic acid anhydrous ethanol solution (1.8 mM, 1.5 mL), H_2_O_2_ (0.03%, 100 μL) were mixed in a test tube at 37 °C water bath for 30 min; Blank group: sample (1 mL), water (2 mL), absolute ethanol (1.5 mL), water (100 μL) were mixed in a test tube then water bath at 37 °C water bath for 30 min. Calibrate the background with a mixture of water (3.1 mL) and absolute ethanol (1.5 mL), measuring the absorbance at 510 nm UV. The clearance rate is calculated as follows:

$${\rm{Clearance}}\,{\rm{Rate}}( \% )=(1-\frac{A1-A2}{A3})\times 100$$

A1 is the absorbance value of the experimental group, A2 is the absorbance value of the blank group, and A3 is the absorbance value of the control group.

### H_2_O_2_ removal test

Control: sample (200 μg/mL, 2 mL), water (2 mL), water (10 μL) reacted for 30 min, then added DTNB (0.01 M, 75 μL), reacted for another 30 min;Experimental group: sample (200 μg/ml, 2 mL), water (2 mL), H_2_O_2_ (0.9 M, 10 μL) reacted for 30 min, then added DTNB (0.01 M, 75 μL), reacted for another 30 min. The background was calibrated with pure water, measured the absorbance at 412 nm UV.

### O_2_·^−^ removal test

The experimental operation is as follows: Solution A: Tris (9.5 g) was dissolved in hydrochloric acid (0.1 mol/L, 12 mL), and TEMED (0.1 mL) was added to make a volume to 25 mL.Solution B: Ammonium persulfate (0.28 g) is dissolved in a small amount of water and then adjusted to 100 mL. Control group: A solution (0.5 mL), B solution (1.5 mL), water (2 mL) reacted for 30 min, then added XTT (0.08 M, 1 mL) reacted for 5 min; Experimental group: A solution (0.5 mL), B solution (1.5 mL), sample (200 μg/mL, 1 mL) reacted for 30 min, then added XTT (0.08 M, 1 mL) reacted for 5 min; Blank group: water (2 mL), sample (200 μg/mL, 1 mL) reacted for 30 min, then added XTT (0.08 M, 1 mL) reacted for 5 min. The background was calibrated with pure water measured the absorbance at 470 nm UV.

## Evaluation of Biocompatibility

### Cytotoxicity experiment

The concentration of S,Se-CQDs in the test were 0 μg/mL, 25 μg/mL, 50 μg/mL, 100 μg/mL, 200 μg/mL and 400 μg/mL, respectively. Cell viability was determined after 24 hours of incubation.

### Zebrafish experiment

Design 6 experimental groups with different concentrations of S,Se-CQDs: 1 μg/mL, 10 μg/mL, 25 μg/mL, 50 μg/mL, 100 μg/mL and 200 μg/mL. 40 fertilized zebrafish eggs per group. Counting the hatching rate of zebrafish eggs incubated with S,Se-CQDs solution (100 μL) after 72 h and the heart rate, survival rate of zebrafish embryo after 96 h. (Experiments on the zebrafishs in this project were in accordance with the protocols approved by the Center of Laboratory Animals Ethics Committee of Guangdong Pharmaceutical University (permit No.: SYXK(YUE)−2012–0125) and the “Guide for the Care and Use of Laboratory Animals” by the National Academy of Sciences (NIH publications No. 80–23, revised 1996).
